# Evaluation of an education and activation programme to prevent chronic shoulder complaints: design of an RCT [ISRCTN71777817]

**DOI:** 10.1186/1471-2296-6-7

**Published:** 2005-02-16

**Authors:** Camiel De Bruijn, Rob de Bie, Jacques Geraets, Marielle Goossens, Albère Köke, Wim van den Heuvel, Geert van der Heijden, Geert-Jan Dinant

**Affiliations:** 1 Institute for Rehabilitation Research, Hoensbroek, The Netherlands; 2 Department of General Practice and Care and Public Health Research Institute, Maastricht University, The Netherlands; 3 Department of Epidemiology, Maastricht University, The Netherlands; 4 Department of Medical, Clinical and Experimental Psychology, Maastricht University, The Netherlands; 5 Hoensbroek Rehabilitation Centre, Hoensbroek, The Netherlands; 6 Julius Center for Health Sciences and Primary Care, University Medical Centre, Utrecht, The Netherlands; 7 Pain Management and Research Centre, University Hospital Maastricht, The Netherlands

## Abstract

**Background:**

About half of all newly presented episodes of shoulder complaints (SC) in general practice are reported to last for at least six months. Early interventions aimed at the psychological and social determinants of SC are not common in general practice, although such interventions might prevent the development of chronic SC.

The Education and Activation Programme (EAP) consists of an educational part and a time-contingent activation part. The aim of the EAP is to provide patients with the proper cognitions by means of education, and to stimulate adequate behaviour through advice on activities of daily living.

**Design:**

The article describes the design of a randomised clinical trial (RCT) to evaluate the effectiveness and cost-effectiveness of an EAP in addition to usual care, compared to usual care only, in the prevention of chronic SC after six months. It also describes the analysis of the cost and effect balance. Patients suffering from SC for less than three months are recruited in general practice and through open recruitment. A trained general practitioner or a trained therapist administers the EAP.

Primary outcome measures are patient-perceived recovery, measured by self-assessment on a seven-point scale, and functional limitations in activities of daily living. Questionnaires are used to study baseline measures, prognostic measures, process measures and outcome measures.

**Discussion:**

The inclusion of patients in the study lasted until December 31^st ^2003. Data collection is to end in June 2004.

## Background

### Shoulder complaints

Shoulder complaints (SC) have been defined by Sobel & Winters [1] as pain localised in the region of the deltoid muscle, the acromioclavicular joint, the superior part of the trapezoid muscle and the scapula. Radiation of the pain to the arm as well as limitation of the motion of the upper arm and/or the shoulder girdle may be present [1].

**Figure 1:**
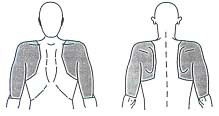
Area between the base of the neck and the elbow

SC are characterised by pain in the area between the base of the neck and the elbow, at rest or when elicited by movement of the upper arm (Fig. 1).

Musculoskeletal disorders, of which SC constitute the second largest group after low back disorders, account for the second largest share in healthcare costs and represent the largest group of work-related diseases in the Netherlands [2].

### Shoulder complaints in general practice

The point prevalence of SC in the general population in the Netherlands has recently been estimated at 21% [3]. In a British study a lower point prevalence of 14% has been found [4]. The annual incidence of SC as seen by general practitioners (GPs) in the Netherlands lies between 15 and 25 patients per 1000 registered general practice patients[1]. About half of all newly presented episodes in general practice are reported to last for at least six months, while 40 percent of the newly presented episodes result in disability in terms of activities of daily living after one year [5].

The International Association for the Study of Pain (IASP) regards persistent or recurring pain lasting less than three months as acute pain, whereas more than three months of persistent or recurring pain is considered to be chronic pain [6]. The study by van der Windt [5] showed that, according to this cut-off criterion, 51% of patients with a newly presented episode of SC in general practice develop chronic SC, that is, complaints lasting more than three months.

The Dutch College of General Practitioners provides clinical guidelines for the treatment of SC [7]. These guidelines, however, do not include treatment aimed at psychosocial factors such as maladaptive behaviour and inadequate cognitions, known to play a role in the development and persistence of chronic musculoskeletal diseases [8-10]. Treatments addressing such factors are mentioned in the guidelines, but only as a last resort, when the biomedical approach has proved ineffective in reducing the pain.

To date, early interventions aimed at the psychological and social determinants of SC are not common in general practice, although such interventions in the early stages of the SC might prevent the development of chronic complaints [11].

### Early intervention in general practice

We hypothesised that an intervention concentrating on psychological and social determinants in the early stages of SC would prevent the development of inadequate cognitions and maladaptive behaviour, ensuring that such inadequate cognitions and behaviour do not play a role in the development of chronic SC later on.

The term cognitions refers to the way patients think about their pain and what the pain means to them, in terms of thoughts, beliefs, attitudes and self-efficacy expectations [12], whereas behaviour refers to the patients' observable actions [13].

Patients' cognitions and behaviour should thus be influenced so as to become adequate cognitions and adaptive behaviour. A relatively brief treatment in the early stages of SC, administered by a trained therapist, may be expected to be effective in preventing the development of chronic SC. The Education and Activation Programme (EAP) that formed the subject of the present study is such an early intervention.

### Aim of the study

This paper describes the design of a study to evaluate the clinical effectiveness of an early EAP aimed at using psychological and behavioural factors to prevent chronic SC. In addition, the study is to evaluate the balance between costs and effects. EAP in patients with acute SC is to be compared with treatment according to the Dutch College of General Practitioners guidelines. The Medical Ethics Committee of the Institute for Rehabilitations Research in association with Rehabilitation Foundation Limburg has approved the design of the study presented here. Funding was obtained from the Netherlands Organisation for Scientific Research.

This paper describes also the rationale and content of the EAP.

### Education and activation programme

Previous studies have indicated that cognitive behavioural therapies aimed at bio-psychosocial factors are promising instruments for the prevention of chronic musculoskeletal pain [14]. The EAP focuses on the same elements as cognitive behavioural therapies, but is applied at an earlier stage than such therapies [15]. Whereas the latter focus mainly on the elimination of inadequate cognitions and maladaptive behaviour after they have already developed in the course of the SC, the EAP focuses on guiding the patient towards adequate pain behaviour and reinforcing this behaviour at an early stage of the SC.

The aim of the EAP is to prevent the development of inadequate cognitions and maladaptive behaviour in patients with acute SC.

Education is used to maintain or induce adequate cognitions by providing information that is tailored to questions that patients have about their SC. Health care educators frequently assume that giving information equals comprehension, which should automatically translate into changed behaviours as the knowledge is applied [16]. According to Hussey, however, simply receiving a message hardly correlates with understanding it [17]. Effective learning also requires the active participation of the patient[18].

Since inadequate cognitions and maladaptive behaviours are not yet fully developed in patients with acute SC, the focus of the EAP is not on restructuring inadequate cognitions or modifying maladaptive behaviour, but on maintaining or inducing the proper cognitions by education and on maintaining or inducing adequate behaviour by giving advice on activities of daily living. Adequate behaviour is considered to be behaviour in which the patient remains active. A comprehensive description of the EAP is given in the Design section.

## Design

### Patients

Patients are recruited by GPs and in the open population by advertising in local newspapers.

Patients are eligible for inclusion in the Randomised Clinical Trial (RCT) if they consult their own GP or respond to adverts in a local newspaper with a new episode of SC that has lasted no longer than three months, at rest or when elicited by movement in the shoulder area.

Patients are included if they are 18 years or older and living in the south of the Netherlands. Only newly presented episodes of SC are considered, that is, patients who have not consulted their GP and have not been treated for their SC in the preceding three months. Additional exclusion criteria are given in table 1.

**Table 1:** Exclusion criteria

• other episodes of SC in the 12 months preceding the consultation with the GP
• prior fractures and/or surgery of the shoulder
• (suspected) referred pain from internal organs
• SC with a confirmed extrinsic cause
• inability to complete a questionnaire independently
• presence of dementia or other severe psychiatric abnormalities

### Randomised Clinical Trial

A Randomised Clinical Trial (RCT) with a six-month follow-up is used to evaluate the effectiveness and cost-effectiveness of an EAP to prevent chronicity in patients with acute SC, compared to usual care.

A computer-generated random sequence table is used to randomise the patients to EAP or usual care. Neither the patient nor the GP, nor the trained therapist, can be blinded for the allocated treatment. The trained therapist is also the researcher coordinating the RCT and conducting the data analysis, but is blinded for treatment allocation during the data analysis. The allocation code will be revealed only after the data analysis has been completed.

### Treatments

Usual care (UC) is applied according to the Dutch College of General Practitioners guidelines for SC (version 1999)[19]. Management during the first two weeks consists of a wait-and-see policy with information and advice about shoulder complaints, possibly supplemented with analgesics or nonsteroidal anti-inflammatory drugs. If this approach has little or no effect, up to three corticosteroid injections can be given. Physiotherapy is considered for complaints persisting after six weeks or more. If the SC persist, referral to a hospital-based specialist may be considered.

The focus of the EAP is to maintain or induce the proper cognitions by education and to stimulate adequate behaviour by means of advice on activities of daily living. Table 2 shows the components of the EAP. The EAP is administered by specially trained GPs or an ambulant therapist (CDB) trained to provide the EAP. The ambulant therapist administers the EAP when no trained GP is available in the living area of the patient.

**Table 2:** Elements of the education and activation programme

**Education**	• Information on the origin, nature and prognosis of the SC
	• Information on possible interventions and their effects (tailored to the patient's questions and needs)
	• Information on the effect of cognitions and behaviour on the perpetuation of the SC
**Activation**	When no alterations in activities have occurred due to the SC
	• Positive reinforcement
	• Instruction to be aware of possible changes
	When alterations in activities have occurred due to the SC
	• Identification of up to three altered frequent activities of daily living
	• Determination of the desired level of activity and the size of the steps needed to reach this level

The EAP consists of a minimum of two sessions and a maximum of six follow-up sessions over a period of six weeks. Each session may last up to 20 minutes. The first and second sessions are organised in the general practice setting by the trained GP, or at the patient's home by the ambulant EAP therapist. The other sessions are provided by telephone.

#### Education

The first part of the EAP has an educational purpose, and focuses on information about the origin, nature and prognosis of the SC, possible interventions and their effects, the impact on activities of daily living and its consequences and the patient's own possibility to contribute to recovery. This information is tailored to the patients' questions and needs and is based on the information available in the Dutch College of General Practitioners guidelines for SC.

In addition, the effect of cognitions and behaviour on the perpetuation of the SC is clarified to the patient by an example. If possible, this example refers to a condition or circumstance the patient has experienced, such as a broken bone or back pain. The patient is helped by the trained GP or the trained therapist to explore whether his or her thoughts about the SC are justified. Negative patterns of thinking are modified into adequate and accurate thoughts.

#### Activation

The second part of the EAP consists of a time contingent activation programme, based on the principles of operant learning. It focuses on gradually increasing activities of daily living, despite the pain.

Potential avoidance of activities is countered by reinforcement of continuation or resumption of usual activities.

Positive reinforcement is used to stimulate patients with a normal activity pattern, in spite of their SC, to continue their activities. This positive reinforcement may be enough to achieve continuation of the desired activities [13]. These patients are also instructed to be aware of possible changes in their activities that could lead to undesirable behaviour such as reduced use of the affected shoulder.

Patients who have reduced their normal activities are helped to identify up to three frequent activities of daily living that they have reduced as a result of the SC. These activities are stepwise gradually increased to the desired level of activity in a time-contingent manner. The desired level of activity and the magnitude of the increases are determined and agreed upon by the EAP therapist and the patient.

The patient and the EAP therapist also plan a progress evaluation, which is used to positively reinforce the patient's behaviour if the gradual increase has been correctly implemented or to adjust the magnitude of the increases if the original objectives prove too optimistic.

### Measurements

The first outcome measure is the perceived recovery of the patient. Patients are considered to be recovered when they report to be much improved or fully recovered, on an 7-point ordinal scale, after six months.

The second outcome measure is that of functional limitations in activities of daily living. This variable is assessed by a 16-item questionnaire, the shoulder disability questionnaire (SDQ)[20], with a scoring range of 0 to 16. A reduction of the score on this questionnaire implies a reduction in functional limitations. The outcome measures are recorded at 6, 12 and 26 weeks after randomisation. The SDQ is also measured at baseline. A cost diary [21] is used to assess health care utilisation, direct non-medical costs and indirect costs. A complete overview of baseline measures, prognostic measures, process measures and outcome measures is given in table 3.

**Table 3:** Variables

**Baseline measures T = 0**	Demographic variables
	• Age
	• Gender
	• Employment status
	Specific disease characteristics
	• Affected side
	• Possible cause of shoulder complaints
	• Duration of complaints
	• History of shoulder complaints
	Co-morbidity
	Physical activity
	Workload
	Treatment credibility and preference
**Prognostic measures T = 0**	Mobility of glenohumeral joint
	• HIB (hand in back), HIN (hand in neck), passive exorotation
	• Active and passive abduction
	Mobility of cervicothoracal spine
	Severity of main complaint
	Psychosocial variables
	• Anxiety^1^
	• Depression^1^
	• Somatisation^1^
	• Distress^1^
	Job content

**Outcome measures T = 1,2,3**	Perceived recovery of complaints Functional limitations to daily activities^2^

**Process measures T = 0,1,2,3**	Psychosocial variables
	• Kinesiophobia^3^
	• Fear avoidance and beliefs^4^
	• Catastrophising^5^
	• Coping with pain^5^
	• Internal locus of control^5^
	• External locus of control^5^
	Global assessment
	Shoulder pain^6^
	General health^7^

**Cost T = 0–26 weeks**	Health care utilisation^8^
	Direct non-medical costs
	Indirect costs

^1 ^four-dimensional complaint list [25]

^2 ^Shoulder Disability Questionnaire [20]

^3 ^Tampa Scale for Kinesiophobia – Dutch version (partly) [26]

^4 ^Fear Avoidance and Beliefs Questionnaire – Dutch version (partly) [27]

^5 ^Pain Coping and Cognition List [28]

^6 ^Shoulder Pain Score [29]

^7 ^Generic Health Related Quality of Life [30]

^8 ^Cost Diary [21]

### Data analysis

The statistical analysis will be carried out according to the 'intention-to-treat' principle. Differences between groups, with 95% confidence intervals, will be calculated for each outcome measure. The study groups will be compared by an independent samples t-test for changes since baseline for continuous outcome variables and the chi-square test for categorical outcome variables. In addition, the corresponding baseline value for each continuous outcome will be used as a covariate.

The analysis will be repeated taking any loss-to-follow-up into account by applying a sensitivity analysis in which all patients who are lost to follow-up are first considered to show the largest observed improvement and then the largest observed deterioration in outcome measures.

The analyses of the difference in change for the outcomes at three and six months will account for the repeated measures character of the data. Baseline characteristics that are a priori considered to be possible prognostic factors for outcome variables, as well as post-randomisation differences between the groups, will be handled as potential confounders. Their influence will be evaluated by means of multivariable regression analyses. In the case of confounding, adjusted effect estimates will also be reported.

### Sample size

About half of all newly presented episodes of SC in general practice are reported to last for at least six months. A number needed to treat of 4.5 after six months is considered clinically relevant. This implies an absolute reduction of 22% of the proportion of patients with SC after six months. With a two-sided alpha of 0.05 and a statistical power (1-β) of 0.80, 70 patients per treatment group are needed to detect a difference in favour of the EAP compared to usual care after six months.

### Embedding in the Dutch Shoulder Disability Study

This RCT is part of the Dutch Shoulder Disability Study, a comprehensive prognostic cohort study on SC, with randomised controlled interventions in subcohorts. The Dutch Shoulder Disability Study is funded by the Netherlands Organisation for Scientific Research (NWO, grant number 904-65-901).

## Discussion

### Reasons for publishing a study design

There are several reasons to publish a study design before the results are available. The main reason is that it provides an opportunity to counteract publication bias, that is, the phenomenon whereby a study producing positive results is more likely to be published than a study showing no difference between the study groups [22], [23]. Hence, if the design is published but not the results, the study can still be included in a systematic review because data can be retrieved from the researcher [24].

Another reason is that it gives researchers the opportunity to reflect upon the study design independently of the results. When results run counter to the researchers' expectations, methodological flaws are usually examined. But when the results are in line with expectations, methodological flaws are more likely to be overlooked. [22]

The third reason arises from the tendency among randomised controlled studies to deviate from their original designs, mainly because of practical problems. Such deviations from the study design may affect the study results. Publishing the study design forces researchers to test its implementation and to answer for any deviations from the design.

Finally, this article offers us an opportunity to describe the rationale and content of the intervention in greater detail than the methods section of an article reporting the results of the RCT would do [24].

### Applicability in general practice

The EAP is a brief intervention that can easily be administered by GPs in addition to the usual care according to the guidelines. This might give GPs an instrument to prevent the development of chronic SC in the early stages of the complaints by focusing on psychological and social determinants.

### Time schedule

The inclusion of patients in the study lasted until December 31^st ^2003. Data collection will be completed in June 2004. Currently, 108 patients have been included and are being followed up.

## Abbreviations

SC: Shoulder Complaints

GP: General Practitioner

IASP: International Association for the Study of Pain

EAP: Education and Activation Programme

RCT: Randomised Clinical Trial

UC: Usual Care

CDB: Camiel De Bruijn

SDQ: Shoulder Disability Questionnaire

NWO: Netherlands Organisation for Scientific Research

## Competing interests

The author(s) declare that they have no competing interests.

## Authors' contributions

CDB participated in the design of the study, will provide the EAP as an ambulant therapist, coordinate the data collection, will perform the statistical analysis and publish the results. RDB participated in the design of the study and will participate in the statistical analysis. JG, MG, WVDH and G-JD also participated in the design of the study. AK participated in the development of the education and activation programme and will conduct the training of the general practitioners that will give the education and activation programme. GVDH conceived of the study, and participated in its design. All authors read and approved the final manuscript.

## Pre-publication history

The pre-publication history for this paper can be accessed here:


